# Classifying Glioblastoma Multiforme Follow-Up Progressive vs. Responsive Forms Using Multi-Parametric MRI Features

**DOI:** 10.3389/fnins.2016.00615

**Published:** 2017-01-11

**Authors:** Adrian Ion-Mărgineanu, Sofie Van Cauter, Diana M. Sima, Frederik Maes, Stefan Sunaert, Uwe Himmelreich, Sabine Van Huffel

**Affiliations:** ^1^Department of Electrical Engineering (ESAT), Signal Processing and Data Analytics, STADIUS Center for Dynamical Systems, KU LeuvenLeuven, Belgium; ^2^imecLeuven, Belgium; ^3^Department of Radiology, University Hospitals of LeuvenLeuven, Belgium; ^4^Department of Electrical Engineering (ESAT), PSI Center for Processing Speech and Images, KU LeuvenLeuven, Belgium; ^5^Department of Imaging and Pathology, Biomedical MRI/MoSAIC, KU LeuvenLeuven, Belgium

**Keywords:** magnetic resonance imaging, multi-parametric, boosting classifiers, glioblastoma multiforme, follow-up

## Abstract

**Purpose:** The purpose of this paper is discriminating between tumor progression and response to treatment based on follow-up multi-parametric magnetic resonance imaging (MRI) data retrieved from glioblastoma multiforme (GBM) patients.

**Materials and Methods:** Multi-parametric MRI data consisting of conventional MRI (cMRI) and advanced MRI [i.e., perfusion weighted MRI (PWI) and diffusion kurtosis MRI (DKI)] were acquired from 29 GBM patients treated with adjuvant therapy after surgery. We propose an automatic pipeline for processing advanced MRI data and extracting intensity-based histogram features and 3-D texture features using manually and semi-manually delineated regions of interest (ROIs). Classifiers are trained using a leave-one-patient-out cross validation scheme on complete MRI data. Balanced accuracy rate (BAR)–values are computed and compared between different ROIs, MR modalities, and classifiers, using non-parametric multiple comparison tests.

**Results:** Maximum BAR–values using manual delineations are 0.956, 0.85, 0.879, and 0.932, for cMRI, PWI, DKI, and all three MRI modalities combined, respectively. Maximum BAR–values using semi-manual delineations are 0.932, 0.894, 0.885, and 0.947, for cMRI, PWI, DKI, and all three MR modalities combined, respectively. After statistical testing using Kruskal-Wallis and *post-hoc* Dunn-Šidák analysis we conclude that training a RUSBoost classifier on features extracted using semi-manual delineations on cMRI or on all MRI modalities combined performs best.

**Conclusions:** We present two main conclusions: (1) using T1 post-contrast (T1pc) features extracted from manual total delineations, AdaBoost achieves the highest BAR–value, 0.956; (2) using T1pc-average, T1pc-90th percentile, and Cerebral Blood Volume (CBV) 90th percentile extracted from semi-manually delineated contrast enhancing ROIs, SVM-rbf, and RUSBoost achieve BAR–values of 0.947 and 0.932, respectively. Our findings show that AdaBoost, SVM-rbf, and RUSBoost trained on T1pc and CBV features can differentiate progressive from responsive GBM patients with very high accuracy.

## 1. Introduction

Glioblastoma multiforme (GBM) is the most common and malignant intracranial tumor (Burger et al., 1985), representing as much as 30% of primary brain tumors with increasing incidence in some geographic regions (Dobes et al., 2011). The patients have a median survival of only 10–14 months after diagnosis with only 3–5% of patients surviving more than three years. Recurrence is universal, and at the time of relapse, the median survival is only 5–7 months despite therapy (Rulseh et al., 2012). The current standard of care is surgical resection followed by radiotherapy and concomitant adjuvant temozolomide chemotherapy (Stupp et al., 2005). Magnetic resonance imaging (MRI) is the most widely used medical imaging technique for identifying the location and size of brain tumors. However, conventional MRI (cMRI) has a limited specificity in determining the underlying type and grade of the brain tumor (Earnest et al., 1988; Dean et al., 1990). More advanced MR techniques like perfusion weighted MRI (PWI) and diffusion kurtosis MRI (DKI) are promising in the characterization of brain tumors as they give potentially more physiological information (Nelson and Cha, 2003; Rees, 2003; Vrabec et al., 2011). DKI visualize the tissue structure and are useful for assessing tumor cellularity, as it gives information about the water movement inside different tissues including biological barriers. Typical parameters related to diffusion are fractional anisotropy (FA), mean diffusivity (MD), and mean kurtosis (MK). FA is a general index of anisotropy, with a value of zero corresponding to isotropic diffusion and a value of one corresponding to diffusion only in one direction. MD is also a general parameter that accounts for the mean diffusivity in all directions, while MK might be a specific parameter for tissue structure (Jensen et al., 2005). PWI provides measurements that reflect changes in blood flow and blood volume. Hypervascularity due to glioma-induced neoangiogenesis may show up as high relative cerebral blood volume (rCBV) while necrosis of different tissues may show up as low rCBV (Lund et al., 2005).

We studied patients with GBM that had their tumor surgically removed and afterwards were treated according to two different protocols developed for evaluating dendritic cell immuno-therapy: HGG-IMMUNO-2003 (De Vleeschouwer et al., 2004, 2008; Rutkowski et al., 2004; Van Gool et al., 2009), and HGG-IMMUNO-2010 (Van Gool et al., 2009).

The focus of this paper is the same as the focus of our previous paper (Ion-Margineanu et al., 2015): finding a map between multi-parametric MRI data acquired during the follow-up of GBM patients and the relapse of brain tumor after surgery, as described by the clinically accepted Response Assessment in Neuro-Oncology (RANO) criteria (Wen et al., 2010). We were motivated to conduct this study because of our excellent previous results where we could differentiate, based only on PWI features, between progressive and responsive follow-up GBM patients with 100% accuracy one month before the patients were labelled according to the RANO criteria. A major drawback of our previous results was the small sample size (18 patients, 27 data points). In this study, we want to confirm our findings on an extended dataset of 29 patients, which includes data from the previous study.

Additionally, we present two main improvements: (1) semi-manually delineating contrast enhancing region (CER) and non-enhancing region (NER) and (2) extracting additional texture and histogram features, with the purpose of improving classification performance. The first improvement is to automatically delineate CER, based on the manually delineated total tumor region. Delineating CER by hand is a time consuming process and requires the radiologist's full attention to make sure that necrosis or non-enhancing regions are not in CER. We select CER based on the T1pc main property of imaging the contrast agent's leakage into the active tumor, which determines high intensity areas where the active tumor is located. The second improvement is extracting histogram and texture features and selecting those with high differentiating power. In the previous paper, we used only the average parameter values from CER, NER, and total. In this paper, we extract six histogram features and 20 3-D texture features based on the gray level co-occurrence matrix (GLCM), as described in Section 2.2.6. We do feature selection using six of the most widely known features selection algorithms and combine feature rankings using the rank product method, as described in Section 2.2.8. In Section 3, linear and non-linear classifiers are tested on a varying number of features, and their results are combined into separate groups, which are used as input to non-parametric statistical tests to discover which combination of delineation, MR modality, and classifier, achieves the highest rank.

## 2. Materials and methods

### 2.1. Study setup

Twenty-nine patients were included in this study, out of which sixteen patients were treated according to the HGG-IMMUNO-2003 protocol (De Vleeschouwer et al., 2004, 2008; Rutkowski et al., 2004; Van Gool et al., 2009), and 13 patients were treated according to the HGG-IMMUNO-2010 protocol (Van Gool et al., 2009; Ardon et al., 2010). Patients treated according to the HGG-IMMUNO-2003 protocol had relapsed GBM and received immunotherapy as the sole treatment strategy. Patients treated according to the HGG-IMMUNO-2010 protocol had primary GBM and underwent surgery. For the follow up treatment after surgery they were split in a double blind placebo controlled randomized clinical trial in which immunotherapy is integrated with radiochemotherapy. At the beginning all patients were scanned on a monthly basis, but after 6 months under immunotherapy they were scanned once every 3 months. The institutional human ethics review board of the University Hospitals of Leuven (Leuven, Belgium) approved this study. Written informed consent was obtained from every patient before participation.

Based on radiological evaluation of the follow-up MRI scans using the current guidelines for response assessment of high grade glioma (Wen et al., 2010), each patient was assigned to one of two clinical groups:

- Patients with **progressive disease** during follow-up which exhibit an increase of ≥25% in the sum of the products of perpendicular diameter of enhancing lesions compared to the smallest tumor measurement obtained either at baseline or best response.
- Patients with **complete response** with disappearance of all measurable and non-measurable disease sustained for at least 4 weeks.

Based on this assessment, each MRI scan of each patient was considered labeled or unlabeled as follows: labeled as “responsive” for all time-points at and after the moment when the patient was considered “complete response;” labeled as “progressive” for all time-points at and after the moment when the patient was considered “progressive disease;” “unlabeled” for all time-points preceding the decision moment. In total there are 183 time points, 56 are labeled and 127 are unlabeled.

### 2.2. MRI acquisition and processing

The MR images were acquired on a clinical 3 Tesla MR imaging system (Philips Achieva, Best, Netherlands), using a body coil for transmission and a 32-channel head coil for signal reception. The imaging protocol consisted of cMRI, PWI, and DKI.

#### 2.2.1. Conventional MRI

Four types of conventional MR images were acquired as previously described (Vrabec et al., 2011; Van Cauter et al., 2012, 2014): an axial spin echo T2-weighted MR image [TR/TE: 3000/80 ms, slice/gap: 4/1 mm, field of view (FOV): 230 × 184 mm^2^, turbo factor (TF): 10, acquisition matrix: 400 × 300]; an axial fluid-attenuated inversion recovery (FLAIR) image (TR/TE/IR: 11,000/120/2800 ms, slice/gap: 4/1 mm, acquisition matrix: 240 × 134) and a T1-weighted 3D spoiled gradient echo scan (fast field echo—FFE, TR/TE: 9.7/4.6 ms, flip angle: 8°, turbo field echo factor: 180, acquisition voxel size: 0.98 × 0.98 × 1 mm^3^, 118 contiguous partitions, inversion time: 900 ms) after contrast administration were acquired as high-resolution anatomical reference images.

#### 2.2.2. Perfusion MRI

PWI were obtained using a standard dynamic-susceptibility weighted contrast perfusion MR imaging protocol consisting of a gradient echo-EPI sequence, TR/TE: 1350/30 ms, section thickness/gap: 3/0 mm, dynamic scans: 60, FOV: 200 × 200 mm^2^, matrix: 112 × 109, number of slices: 23, scan time: 1 min 26 s. EPI data were acquired during the first pass following a rapid injection of a 0.1 mmol/kg body weight bolus of megluminegadoterat (Dotarem, Guerbet, Villepinte, France) via a mechanical pump at a rate of 4 ml/s, followed by a 20 ml bolus of saline. Preload dosing was performed according to Hu et al. in order to correct for T1-weighted leakage (preload dose 0.1 mmol/kg megluminegadoterat, incubation time 10 min) (Hu et al., 2010). PWI were processed using the DSCoMAN plugin (Boxerman et al., 2006) for ImageJ (http://rsb.info.nih.gov/ij/), which takes into consideration the leakage correction and can easily be automated. For each PWI acquisition, five parameter maps were extracted: corrected cerebral blood volume (CBV), cerebral blood flow (CBF), mean transit time (MTT), time to peak (TTP), and *R*^2^.

#### 2.2.3. Diffusion MRI

DKI data were acquired according to the previously described protocol in Van Cauter et al. (2012, 2014) SE-EPI-DWI sequence with TR/TE: 3200/90 ms, δ/Δ: 20/48.3 ms; FOV: 240 × 240 mm^2^, matrix: 96 × 96, number of slices: 44, 1 signal average acquired, section thickness/gap: 2.5/0 mm, b-values: 0, 700, 1000, and 2800 s/mm^2^ in 10, 25, 40, and 75 uniformly distributed directions, respectively (Poot et al., 2010). The DKI data were processed as described in Van Cauter et al. (2014). For each DKI acquisition, seven parameters maps were derived from the tensors (Jensen et al., 2005; Hui et al., 2008): fractional anisotropy (FA), mean diffusivity (MD), axial diffusivity (AD), radial diffusivity (RD), mean kurtosis (MK), axial kurtosis (AK), radial kurtosis (RD).

#### 2.2.4. Delineations

A regions of interest (ROI) was manually drawn around the Total tumor lesion, avoiding areas of necrosis, or cystic components such as the surgical cavity. A separate ROI was drawn around the contra-lateral normal appearing white matter (NAWM) to standardize measurements extracted from the tumor region. The Total and NAWM ROIs were drawn by SVC, a radiologist with 5 years experience.

To automatically split the Total region in two ROIs, CER and NER, a threshold was set at the 90th percentile of T1pc Total voxels. In this way, two semi-manual ROIs were made for each patient based on the T1pc intensities selected from Total: CER, containing very high T1pc intensity Total voxels, and NER, containing the rest of Total voxels.The 90th percentile threshold was selected after visually inspecting T1pc maps of multiple patients.

**Figure 1:**
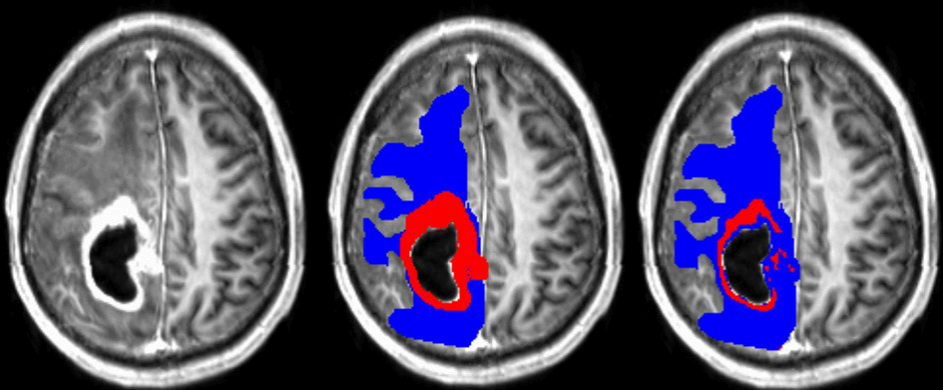
**(Left)** T1pc. **(Center)** Manual delineations on top of T1pc. **(Right)** Semi-manual delineations on top of T1pc. CER is red and NER is blue.

A typical example of manual and semi-manual ROIs on T1pc can be seen in Figure 1, where red is CER and blue is NER.

#### 2.2.5. Co-registration

All four cMRI maps, T1, T1pc, T2, and FLAIR, were first skull-stripped using FSL-BET with default parameters (Smith, 2002). Afterwards, affine co-registration of skull-stripped T1, T2, and FLAIR to skull-stripped T1pc was done using NiftyReg (Ourselin et al., 2002) with default parameters (http://cmictig.cs.ucl.ac.uk/wiki/index.php/NiftyReg). Three affine transformation matrices were saved and used to re-sample the corresponding original T1, T2, and FLAIR to the T1pc space.

To co-register PWI a similar protocol was used. Each PWI scan has 60 T2^*^ brain volumes that can be selected to be co-registered to T1pc. In this study, we select the first PWI brain volume and assume that the rest of them are well-aligned with it, ignoring any artifacts. After skull-stripping the first PWI volume using FSL-BET with default parameters, affine co-registration to skull-stripped T1pc was done using NiftyReg with default parameters. We obtain one affine transformation that is used to co-register all five PWI parameter maps (described in Subsection 2.2.2) to T1pc.

To co-register DKI a similar protocol was used. Each DKI scan has 10 T2 brain volumes that can be selected to be co-registered to T1pc. In this study, we select the first DKI brain volume and assume that the rest of them are well aligned with it, ignoring any artifacts. After skull-stripping the first DKI volume using FSL-BET with default parameters, affine co-registration to skull-stripped T1pc was done using NiftyReg with default parameters. We obtain one affine transformation that is used to co-register all seven DKI parameter maps (described in Subsection 2.2.3) to T1pc.

**Figure 2:**
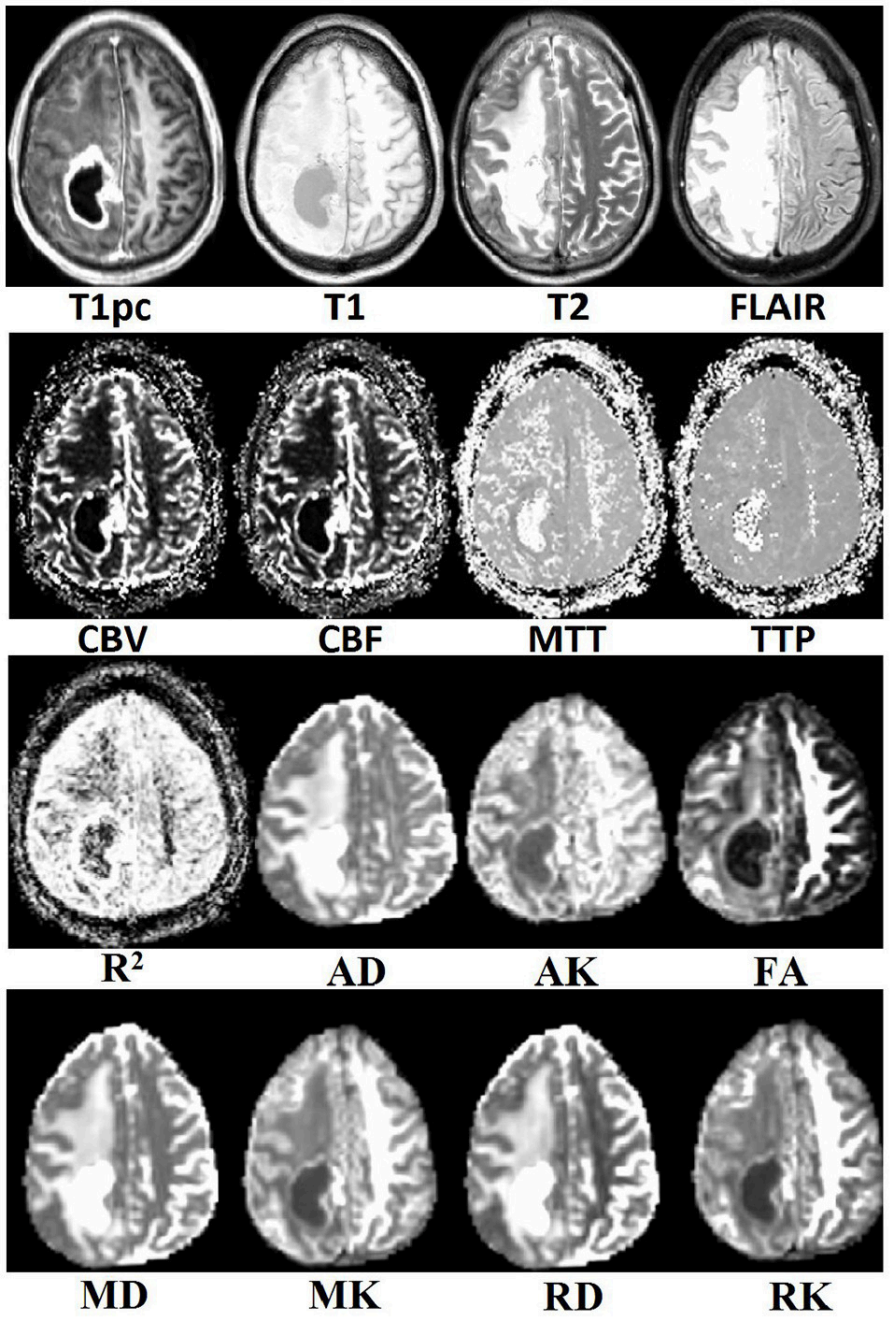
**Example of co-registration results for all multi-parametric MR maps**.

Visual inspection of the tumor's center in the axial plane of all maps for all patients after co-registration to T1pc was done by AIM and found no major misalignments. An example of all 16 maps for a random patient can be seen in Figure 2.

#### 2.2.6. Feature extraction

After co-registering all maps to T1pc, the three ROIs (Total, CER, NER) are used as separate 3-D masks on each map to extract histogram and texture features. On each map, voxel intensities from each mask were normalized to the average value computed from the corresponding NAWM ROI. For each mask six histogram measures are computed: mean, coefficient of variation, 90th percentile, 10th percentile, skewness, and kurtosis. Additionally, 20 texture features are extracted from the 3-D GLCM (Haralick et al., 1973). To compute the GLCM, each map has been rescaled such that the voxel intensities are integers varying from 1 to 64.

The GLCM computation was done using the function *graycomatrix* implemented in Matlab R2015a (MathWorks, Massachusetts, U.S.A.) with distance set to 1, the “*Symmetric*” flag set to *true*, and four values of “*Offset*” set to the four main directions: 0°, 45°, 90°, and 135°. Twenty 3-D texture features, as described in Haralick et al. (1973), Soh and Tsatsoulis (1999), and Clausi (2002), were extracted from GLCM: autocorrelation, contrast, correlation, cluster prominence, cluster shade, dissimilarity, energy, entropy, homogeneity, maximum probability, sum of squares: variance, sum average, sum variance, sum entropy, difference variance, difference entropy, information measure of correlation (IMC) 1 and 2, inverse difference normalized (IDN), and inverse difference moment normalized (IDMN).

In the end 416 features are extracted from each ROI: 26 histogram and texture features for each of the 16 maps. All features have been rescaled between 0 and 1, using all 183 labeled and unlabeled time points. In total, our dataset has 56 data points and 1248 features.

#### 2.2.7. Datasets comparison

In this study we analyze two main influences: (1) features extracted from CER&NER vs. features extracted from Total tumor; (2) cMRI vs. PWI vs. DKI vs. all multi-parametric MRI features (cPD). Therefore, we split the original dataset according to the two main influences and create eight smaller datasets. These eight datasets were built using *complete* labeled time points, meaning labeled time points which have all MRI data available. Only complete time points were selected because in order to have a fair comparison between different MRI modalities, the number of points must not differ between them. All eight datasets have acquisitions from 29 patients and 55 time points. The ratio between progressive and responsive time points is slightly unbalanced, 34–21, or 62 vs. 38%. Table A1 from Appendix I shows the number of features in each dataset.

#### 2.2.8. Feature selection methods

In order to avoid the curse-of-dimensionality (Bellman, 2015), the number of data points should be much larger than the number of features. Because there are only 55 data points, different feature selection methods were used to reduce the dimension between 1 and 10 (Hua et al., 2005). In this study, six of the most widely known feature selection algorithms were used: minimum redundancy maximum relevance (mRMR) (Ding and Peng, 2005), RELIEFF (Kononenko et al., 1997), information gain (InfoGain) (Yang and Pedersen, 1997), Pearson's Chi^2^ (Yang and Pedersen, 1997), random forest—mean decrease in accuracy (RF-MDA) (Breiman, 2001), and random forest—mean decrease in Gini (RF-MDG) (Breiman, 2001; Calle and Urrea, 2011). The first four methods were run using the WEKA (Hall et al., 2009) application program interface (API) in Matlab R2015a. The last two methods were run using a random forest (RF) of 10,000 trees in the statistical environment R (Liaw and Wiener, 2002). Principal component analysis or other dimension reduction methods are not used because the biomedical meaning of the extracted features is lost.

#### 2.2.9. Cross validation and performance measure

Given the fact that multiple data points per patient were acquired from 29 patients, a leave one patient out cross validation (LOPOCV) setup is used. In this way, 29-folds are created in which the test patient is always independent of the training patients: in each fold data points from one patient are considered test points, while data points from the remaining 28 patients are used for training.

In this study, two LOPOCV rounds are done, one for feature selection, and another one for classification using fixed feature sets.

In the first round, feature rankings are learned on the training sets, with the most important features at the top, and the least important at the bottom. Because each fold will have six different feature rankings as outputs from mRMR, Relieff, InfoGain, Chi^2^, RF-MDA, and RF-MDG, each dataset will have 174 feature rankings. Combining different rankings is done by computing rank products (Breitling et al., 2004) of each feature that appears at least once in top 10 of any feature ranking. The output of the first round of LOPOCV is a fixed set of 10 features selected by rank products per dataset shown in Table A4 from Appendix I.

In the second round, increasing number of features from 1 to 10 were used to classify data points. Classifiers are trained on the training set of each fold, then they assign a label to each testing data point from the test set. The assigned labels are compared to the true labels by measuring the balanced accuracy rate (BAR) of all 55 test points. BAR, defined as the average between sensitivity and specificity, was preferred as a performance measure because the interest is in classifying correctly both labels (responsive and progressive). BAR can take values between 0 and 1, 1 pointing to a perfect classification, and 0 to a completely wrong classification. A random classifier should give a BAR–value of 0.5.

### 2.3. Classifiers

Several supervised classifiers have been used, with the goal of testing if data labeled according to the RANO criteria could have been reliably labeled using histogram and 3-D texture measures extracted from multi-parametric MRI. The list of classifiers as well as their software implementation environment is presented in Table A2, Appendix I.

The list of classifiers in Table A2 is representative for most of the classification algorithms, starting from simple linear ones such as Linear Discriminant Analysis (LDA) and Support Vector Machines with linear kernel (SVM-lin) up to more complex non-linear classifiers such as RF and Stochastic Gradient Boosting (SGB).

Fisher's LDA (Fisher, 1936) is a classifier that finds a linear combination of input features that best separates the two classes. It is also very easy to use as there are no parameters that need to be set. Support Vector Machines (Cortes and Vapnik, 1995; Cristianini and Shawe-Taylor, 2000) is among the most popular machine learning models because of its simplicity: given a training set with points from two classes, it tries to find the best hyperplane to differentiate between the two types of points. It can be used in the original feature space or the points can be mapped to another space by using kernel transformations. Two types of SVM kernel were used in this study: linear (SVMlin) and radial basis function (SVMrbf) with default settings (“C” and “sigma” set to 1). Random forests (Breiman, 1996, 2001) are part of the ensemble methods for classification that use a collection of decision trees. Each decision tree learns a rule on a bootstrap sample of the original dataset and then it can classify a new point. The new point is assigned to the class voted by the majority of the trees. In this study, RF was run with 1000 trees on all input features and “class_weight” set to “balanced_subsample,” to adjust weights for each data point inversely proportional to class frequencies for each individual tree. Boosting algorithms (Freund and Schapire, 1995; Seiffert et al., 2008) start with a collection of weak classifiers, in this case decision trees, and with each iteration they try to improve the overall classification by learning what was misclassified at the previous step. In this paper, the boosting algorithms had the following parameters: AdaBoost was run with 1000 trees; SGB was run with 1000 trees, “learning_rate” set to 0.1 (default), and “subsample” set to 0.5, as suggested in Friedman (2002) Random Under Sampling Boosting (RUSBoost) was run with 1000 trees and “LearnRate” set to 0.1.

## 3. Results

We compared seven classifiers, four MR modalities, two types of delineations, with a variable number of features from one to ten, summing up to a total of 560 BAR–values. These BAR–values were grouped in several ways (e.g., 56 groups of 10 values), and then a non-parametric comparison was made to account for statistical differences between groups. Multiple Kruskal and Wallis (1952) rank tests were run in MATLAB R2015a to determine if all groups originate from the same distribution, followed by Dunn-Šidák's *post-hoc* test (Dunn, 1964; Šidák, 1967) to determine which group had the highest rank (better accuracy results). The relationship between BAR–values and ranks is as follows: each BAR–value is assigned a rank value, ignoring group membership. The assigned rank should be an integer, except for the case when there are multiple equal BAR–values, then the assigned rank is the average of the individual ranks. For example, the BAR sequence [0.5, 0.7, 0.7, 0.9] is transformed into the rank sequence [1, 2.5, 2.5, 4].

**Figure 3:**
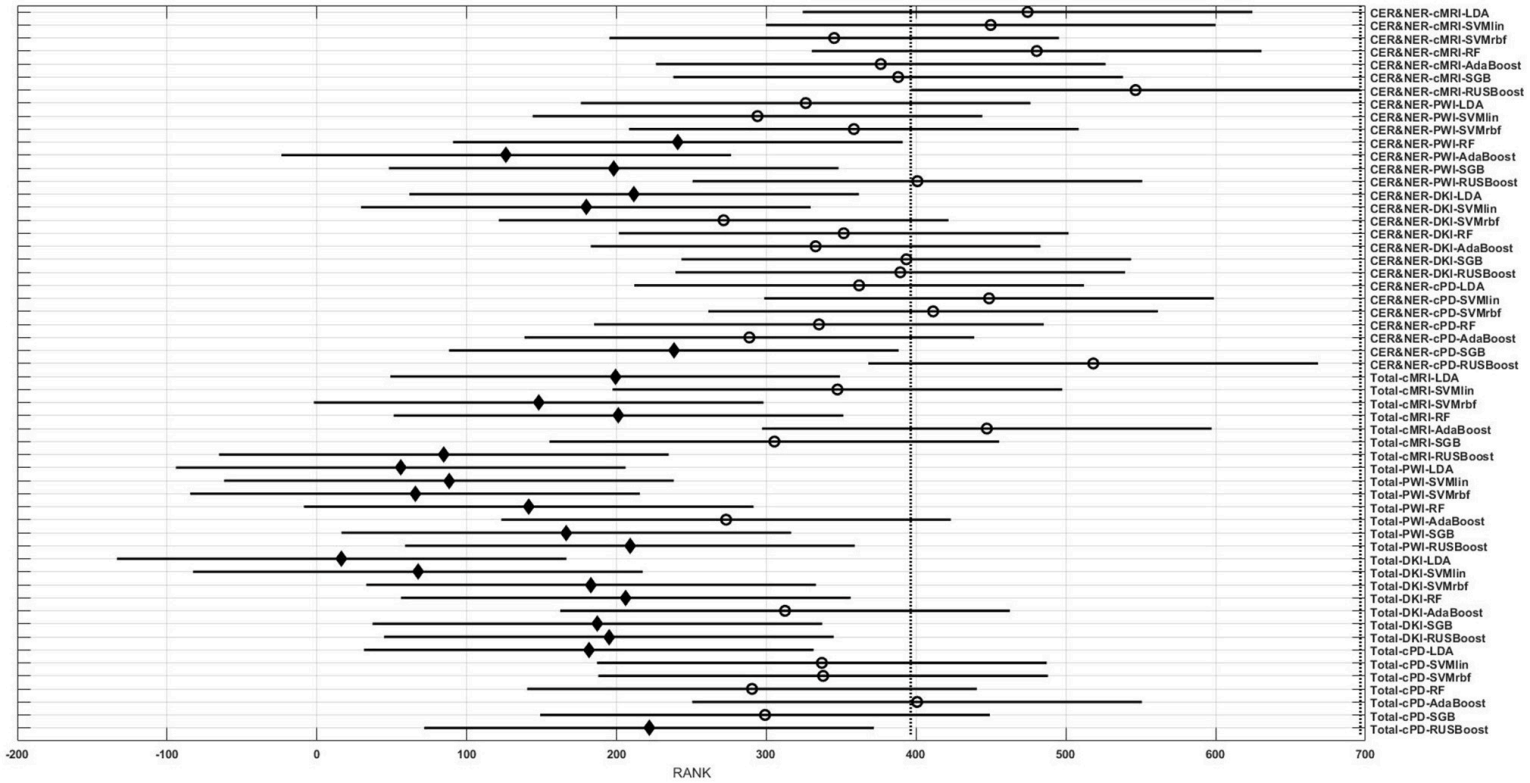
**Rank estimates and intervals for all combinations of classifiers, delineations, and MR modalities**.

**Figure 4:**
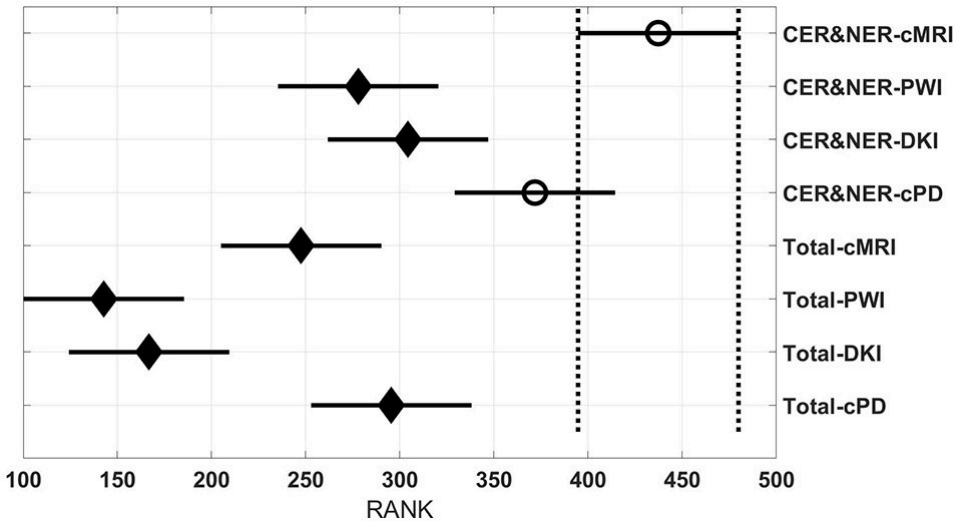
**Rank estimates and intervals for all combinations of delineations and MR modalities**.

**Figure 5:**
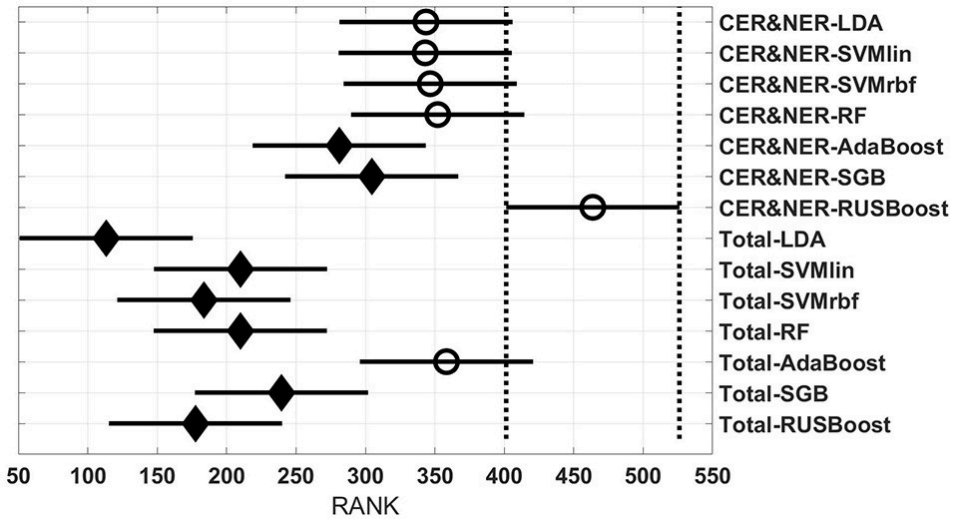
**Rank estimates and intervals for all combinations of delineations and classifiers**.

**Figure 6:**
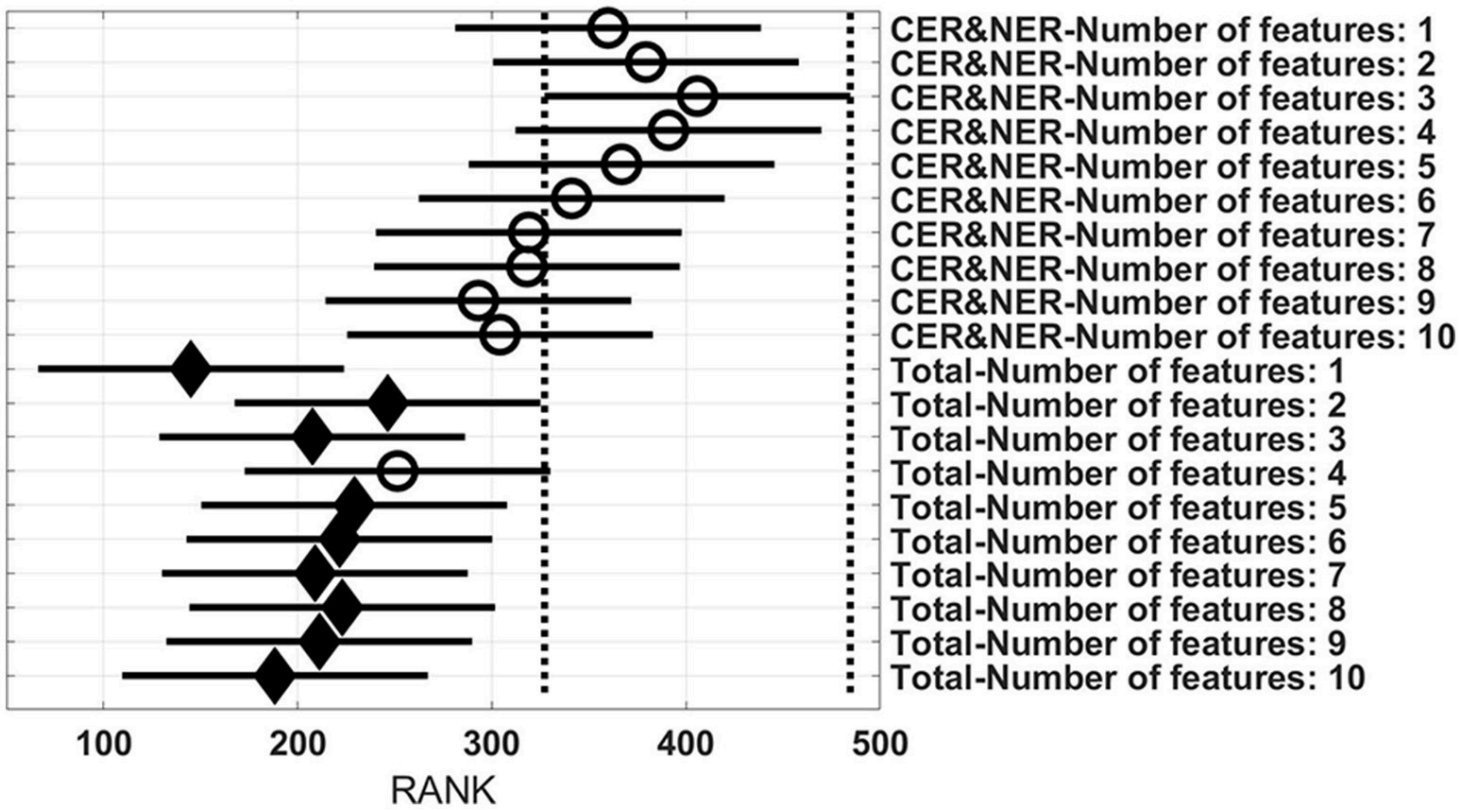
**Rank estimates and intervals for all combinations of delineations and varying number of features**.

Figures 3–**6** show rank estimates and intervals of different groups. Intervals are shown as horizontal lines, while rank estimates are in the middle of the intervals. In each figure, the highest ranked group has its interval limited by two vertical dotted lines. Groups that are significantly different from the highest ranked group have a filled diamond marker in the middle of their interval, while groups that are not significantly different from the highest ranked group have an empty circular marker in the middle of their interval. Two groups are significantly different if their intervals are disjoint; they are not significantly different if their intervals overlap.

Figure 3 shows 56 groups, each group containing 10 BAR–values coming from test runs with varying only the number of features. In the upper part there are 28 groups using CER&NER features, while in the lower part there are 28 groups using Total tumor features. The highest ranked group is CER&NER-cMRI-RUSBoost and its rank is significantly higher than 18 out of 28 groups achieved with Total tumor features.

Figure 4 shows eight groups, each group containing 70 BAR–values coming from test runs with varying classifiers and number of features. In the upper part there are four groups using CER&NER features, while in the lower part there are four groups using Total tumor features. The highest ranked group has CER&NER-cMRI features and its rank is significantly higher than all groups using Total tumor features. Moreover, the CER&NER-cMRI group has a significantly higher rank than CER&NER-PWI and CER&NER-DKI. This means that classification based only on conventional MRI features performs better than the classification based only on perfusion or diffusion features.

Figure 5 shows 14 groups, each group having 40 BAR–values coming from test runs with varying MR modalities and number of features. In the upper part there are seven groups using CER&NER features, while in the lower part there are seven groups using Total tumor features. The highest ranked group is CER&NER-RUSBoost and its rank is significantly higher than all groups using Total tumor features except AdaBoost.

Figure 6 shows 20 groups, each group having 28 BAR–values coming from test runs with varying MR modalities and classifiers. In the upper part there are 10 groups using CER&NER features, while in the lower part there are 10 groups using Total tumor features. The highest ranked group is CER&NER-Number of features:3 and its rank is significantly higher than all groups using Total tumor features except Total-Number of features:4.

**Figure 7:**
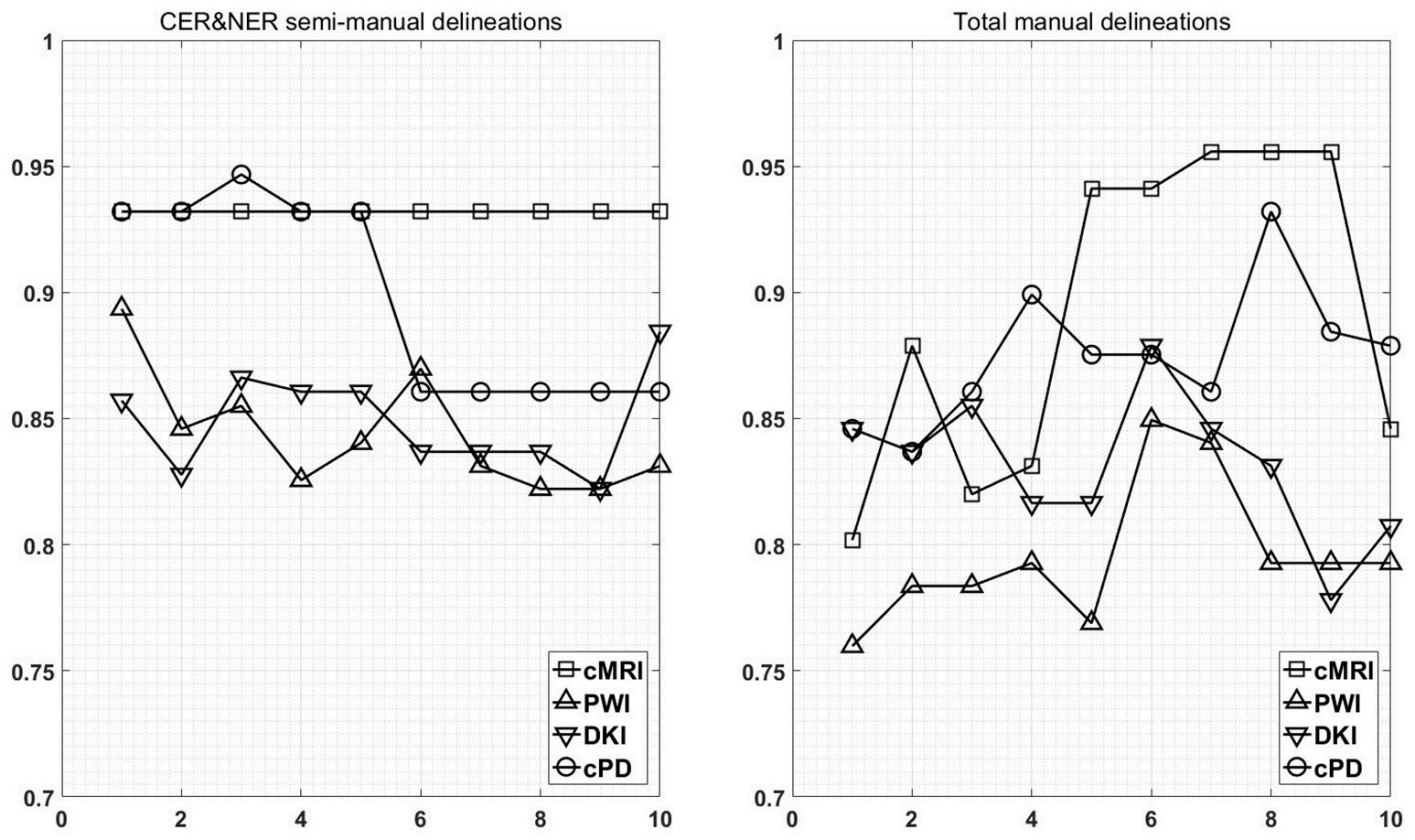
**Maximum classification results over all MR modalities using 1–10 features**. On y-axis are BAR–values, and on x-axis the number of features used for classification.

**Figure A1:**
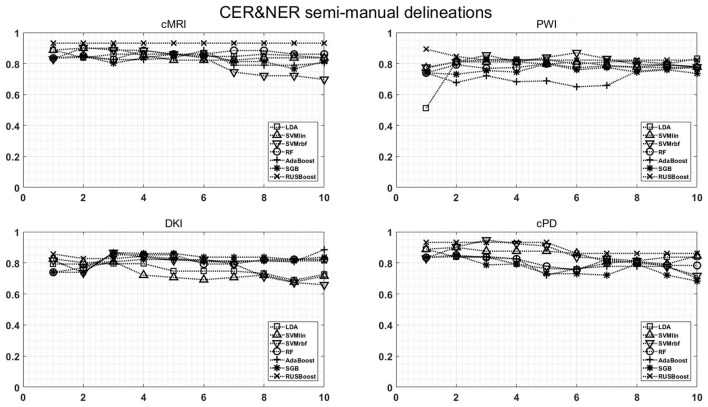
**Classification results on CER & NER semi-manual delineations, using 1–10 features assigned by rank products per each dataset**. On y-axis are BAR–values, and on x-axis the number of features used for classification.

**Figure A2:**
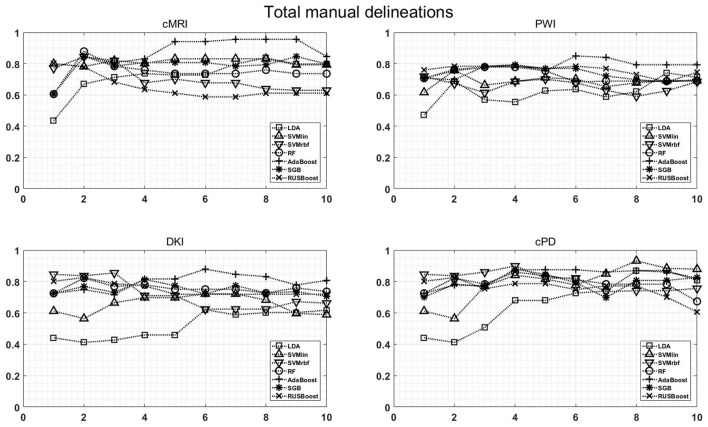
**Classification results on Total manual delineations, using 1–10 features assigned by rank products per each dataset**. On y-axis are BAR–values, and on x-axis the number of features used for classification.

Figure 7 shows the maximum BAR over all MR modalities for CER&NER and Total tumor ROIs, when varying the number of features from 1 to 10. In Appendix I, associated with Figure 3, there is Table A3. Figures A1, A2 from Appendix I show results of each classifier when varying the number of features from 1 to 10 for each MR modality, for CER&NER and Total tumor ROIs, respectively.

Multiple remarks can be made after analyzing the figures and tables previously presented. One of the most important remarks is that there is no combination of features, classifier, and delineations, that gives a perfect BAR–value of 1. Although all *post-hoc* tests show the superiority of features extracted from contrast-enhancing and non-enhancing regions, the highest BAR–value is achieved using total tumor features. To compare, the highest BAR–value obtained using total tumor features is 0.956, with AdaBoost on cMRI, between 7 and 9 features, while the highest BAR–value obtained using contrast-enhanced and non-enhanced features is 0.947, with SVMrbf trained on the first three cPD features. It is interesting to note that the maximum BAR–value using total tumor features is achieved using only T1pc features, while the maximum BAR–value using contrast-enhanced and non-enhanced features is achieved only after combining perfusion features with T1pc features. To be more specific, the maximum BAR–value using contrast-enhanced and non-enhanced features is achieved only after training SVMrbf on the following three features: T1pc-average-CER, T1pc-90th_percentile-CER, and CBV-90th_percentile-CER.

Another notable result is the fact that RUSBoost trained only on just one feature (T1pc-Average-CER) achieves a remarkable BAR–value of 0.932. This BAR–value is maintained by RUSBoost trained on all 10 contrast-enhanced and non-enhanced conventional MRI features, indicating a robust classification. The same BAR–value of 0.932 is achieved by RUSBoost also on the first five CER&NER-cPD features, which are histogram features extracted from T1pc, CBV, and CBF. When we add the sixth cPD feature, a diffusion texture feature (ak-IMC2-CER), the BAR–value drops to 0.86.

The *post-hoc* results reflect the consistent high BAR–values of RUSBoost trained on contrast-enhanced and non-enhanced features, placing it at the two highest ranked positions in Figure 3. Although no group is significantly higher than the rest of groups, CER&NER-cMRI-RUSBoost is ranked significantly higher than most of Total-PWI and Total-DKI classifiers. Top two classifiers using Total tumor features that are not significantly different than CER&NER-cMRI-RUSBoost are Total-AdaBoost-cMRI and Total-AdaBoost-cPD.

Figure 4 shows a surprising result: the best ranked group, compared to all but one, is CER&NER-cMRI. This is surprising because it means that there is no need to acquire perfusion or diffusion MRI, one can obtain high BAR–values using only conventional MRI features. Although we are aware that the highest BAR–value using contrast-enhanced and non-enhanced features was obtained after combining perfusion and conventional MRI features, BAR–values obtained only on CER&NER-PWI or CER&NER-DKI features were ranked significantly lower than CER&NER-cMRI. BAR–values obtained using Total tumor features with any kind of MR modality were also ranked significantly lower than CER&NER-cMRI.

Analyzing Figure 5 we can make two remarks: RUSBoost is the best classifier using contrast-enhanced and non-enhanced features and AdaBoost is the best classifier using total tumor features. Analyzing Figure 6 we see that for CER&NER, increasing the number of features above a threshold of 3 will decrease the BAR–values, although not significantly.

It is clear from these four figures, Figures 3–6 that splitting the total tumor into two regions, contrast-enhancing and non-enhancing, using a simple threshold like 90th percentile, can improve significantly the classification accuracy.

## 4. Discussion

In Table A4 we can see that feature selection for contrast-enhanced and non-enhanced features revealed an interesting result: only one feature was selected from the non-enhancing group (for DKI, 8th position). This means that contrast-enhancing features are very meaningful and we could rely only on them in future works involving classification or statistical analysis.

When comparing the number of features selected from histogram or texture, even though we extracted more texture features (20 compared to 6), we can see a relative balance in all MR modalities except DKI. In top 10 CER&NER-cMRI features, 4 come from histogram; in top 10 CER&NER-PWI features, 4 come from histogram; in top 10 CER&NER-cPD features, 6 come from histogram; in top 10 CER&NER-DKI features, only 1 comes from histogram. In top 10 Total-cMRI features, 4 come from histogram; in top 10 Total-PWI features, 2 come from histogram; in top 10 Total-cPD features, 5 come from histogram; in top 10 Total-DKI features, none come from histogram. These feature rankings strongly suggest that if only DKI data is available, one should definitely extract texture features to assess tumor recurrence.

When selecting cPD features, for both CER&NER and Total tumor ROIs, we can see in top 5 the same two features: T1pc-90th_percentile and CBV-90th_percentile. This selection comes as a confirmation of the majority of literature articles showing that contrast enhancement areas and CBV–values are strongly correlated to tumor progression (Lund et al., 2005; Barajas Jr. et al., 2009; Hu et al., 2012, 2011). The main reason behind this strong correlation is the fact that tumors grow uncontrollably, so they require more nutrients compared to surrounding tissue, which is reflected in the tumor's angiogenesis. The increase in angiogenesis is visualized and measured using T1pc and PWI.

There are multiple studies that focus on predicting the treatment outcome of follow-up GBM patients using multi-parametric MR data. The majority focuses mainly on overall survival, true progression vs. pseudo-progression or true progression vs. radiation injury. Some recent examples are the following Elson et al. (2015) show using DKI data from 52 patients that Apparent Diffusion Coefficient (ADC)–values strongly correlate to overall survival Smets et al. (2013) conclude on 24 patients that absence of contrast enhancement on immediately post-operative T1pc correlates to an increase in overall survival Zhang et al. (2016) developed a new feature selection method using DKI data from 79 patients which gives an area under the receiver operating characteristic (ROC) curve (AUC) of 0.86 for separating true from pseudo-progression, without any manual segmentation; Bulik et al. (2015) found significant differences in ADC and spectroscopic metabolites values between patients with true and pseudo-progression Di Costanzo et al. (2014) show, using data from 29 patients, that LDA trained on ADC, CBV, and normalized Choline gives a 96.6% accuracy in differentiating patients with true progression vs. radiation injury Khalifa et al. (2016) show that the fraction of hypoperfused tumor volume gives a 79.2% accuracy in anticipating tumor relapse at the next follow-up point.

Our study is, to our best knowledge, the only one that tries to classify progressive vs. responsive follow-up GBM patients based on multi-parametric MR data acquired at 3T. In our previous paper we showed, using data from 18 patients, that PWI is a very powerful predictor of tumor recurrence, obtaining 100% accuracy in predicting the label one month before the label was put according to RANO criteria. In this paper, we used data acquired from 29 patients, therefore the classification problem is more difficult because of the increasing overlap between the classes. However, we still obtained maximum BAR–values higher than 0.85 for each dataset: (i) Contrast-enhancing and Non-enhancing features - cMRI-0.932, PWI-0.894, DKI-0.885, cPD-0.947; (ii) Total tumor features—cMRI-0.956, PWI-0.85, DKI-0.879, and cPD-0.932. Although the maximum value is achieved using features extracted from Total tumor ROI and not CER&NER (0.956 vs. 0.947), we showed using non-parametric multiple comparison tests that it is recommended to use features from CER&NER, which could be defined by a simple threshold like the 90th percentile on T1pc-Total ROI.

## 5. Conclusions

We proposed an automatic pipeline for processing multi-parametric MR data acquired at 3T and validated it after extracting histogram and GLCM 3-D texture features. We determined the added value of extracting features from semi-manually delineated contrast-enhancing and non-enhancing ROIs compared to features extracted from manual total tumor ROIs using non-parametric multiple comparison tests. We showed that AdaBoost, RUSBoost, and SVM-rbf trained mainly on features extracted from T1pc and CBV maps achieve the highest ranked performance in classifying progressive vs. responsive follow-up GBM patients. Finally, our results suggest that using only conventional MRI features is better than using only perfusion or diffusion MRI features in the same classification problem.

## Author contributions

AI, DS, FM, and SVH designed the methodological part of the study. SVC, SS, and UH designed the clinical part of the study. SVC collected the data. AI analyzed the data. All authors contributed to the manuscript.

## Funding

This work was funded by the following projects: Flemish Government FWO project G.0869.12N (Tumor imaging); Belgian Federal Science Policy Office: IUAP P7/19/ (DYSCO, “Dynamical systems, control and optimization,” 2012–2017); EU: The research leading to these results has received funding from the European Research Council under the European Union's Seventh Framework Programme (FP7/2007–2013)/ERC Advanced Grant: BIOTENSORS (no. 339804). This paper reflects only the authors' views and the Union is not liable for any use that may be made of the contained information. Other EU funding: EU MC ITN TRANSACT 2012 (no. 316679).

### Conflict of interest statement

The authors declare that the research was conducted in the absence of any commercial or financial relationships that could be construed as a potential conflict of interest.

## Appendix I

**Table A1:** **Number of features per MRI modality and delineation**.

	**CER&NER**				**Total ROI**			
	**cMRI**	**PWI**	**DKI**	**cPD**	**cMRI**	**PWI**	**DKI**	**cPD**
Number of features	208	260	364	832	104	130	182	416

**Table A2:** **Supervised classifiers**.

**Classifiers**	**Software**
Linear Discriminant Analysis (LDA)	Matlab R2015a—Statistics and Machine Learning Toolbox
Support Vector Machine (SVM)	Matlab R2015a—Statistics and Machine Learning Toolbox
Random Forests (RF)	Python 2.7.6—sklearn.ensemble.RandomForestClassifier
Adaptive Boosting (AdaBoost)	Python 2.7.6—sklearn.ensemble.AdaBoostClassifier
Stochastic Gradient Boosting (SGB)	Python 2.7.6—sklearn.ensemble.GradientBoostingClassifier
Random Under Sampling Boosting (RUSBoost)	Matlab R2015a—Statistics and Machine Learning Toolbox

**Table A3:** **Maximum BAR of all MR modalities over all classifiers**.

**Dataset**	**Number of features**	**cMRI**	**PWI**	**DKI**	**cPD**
CER&NER	1	0.9321	0.8936	0.8571	0.9321
	2	0.9321	0.8459	0.8277	0.9321
	3	0.9321	0.8550	0.8662	0.9468
	4	0.9321	0.8256	0.8606	0.9321
	5	0.9321	0.8403	0.8606	0.9321
	6	0.9321	0.8697	0.8368	0.8606
	7	0.9321	0.8312	0.8368	0.8606
	8	0.9321	0.8221	0.8368	0.8606
	9	0.9321	0.8221	0.8221	0.8606
	10	0.9321	0.8312	0.8845	0.8606
Total ROI	1	0.8018	0.7598	0.8459	0.8459
	2	0.8789	0.7836	0.8368	0.8368
	3	0.8200	0.7836	0.8550	0.8606
	4	0.8312	0.7927	0.8165	0.8992
	5	0.9412	0.7689	0.8165	0.8754
	6	0.9412	0.8494	0.8789	0.8754
	7	0.9559	0.8403	0.8459	0.8606
	8	0.9559	0.7927	0.8312	0.9321
	9	0.9559	0.7927	0.7780	0.8845
	10	0.8459	0.7927	0.8074	0.8789

**Table A4:** **Top 10 selected features according to rank products for each dataset**.

**Dataset**	**cMRI**	**PWI**	**DKI**	**cPD**
CER&NER	T1pc-average-CER	CBV-average-CER	ak-IMC2-CER	T1pc-average-CER
	T1pc-90th_percentile-CER	MTT-homogeneity-CER	ak-IMC1-CER	T1pc-90th_percentile-CER
	T1pc-10th_percentile-CER	CBV-90th_percentile-CER	fa-autocorrelation-CER	CBV-90th_percentile-CER
	FLAIR-cluster shade-CER	MTT-IDN-CER	fa-sum variance-CER	CBV-average-CER
	FLAIR-skewness-CER	MTT-maximum probability-CER	fa-sum of squares-CER	CBF-90th_percentile-CER
	FLAIR-sum of squares:variance-CER	CBF-90th_percentile-CER	fa-average-CER	ak-IMC2-CER
	T2-cluster shade-CER	MTT-dissimilarity-CER	fa-sum average-CER	MTT-homogeneity-CER
	FLAIR-autocorrelation-CER	Rsquare-homogeneity-CER	ad-cluster prominence-NER	CBF-average-CER
	FLAIR-sum variance-CER	MTT-difference entropy-CER	mk-IMC2-CER	MTT-IDN-CER
	FLAIR-sum average-CER	CBF-average-CER	mk-IMC1-CER	ak-IMC1-CER
Total ROI	T1pc-90th_percentile	CBV-90th_percentile	ad-cluster prominence	ad-cluster prominence
	T1pc-coefficient of variation	Rsquare-IMC2	md-cluster prominence	md-cluster prominence
	T1pc-cluster shade	Rsquare-IMC1	ad-cluster shade	T1pc-90th_percentile
	T1pc-sum average	CBF-90th_percentile	rd-sum entropy	T1pc-coefficient of variation
	T1pc-skewness	CBV-IMC2	md-cluster shade	CBV-90th_percentile
	T1pc-sum variance	MTT-correlation	md-sum entropy	ad-cluster shade
	T1pc-autocorrelation	CBF-IMC2	rd-cluster prominence	T1pc-skewness
	T1pc-sum of squares:variance	Rsquare-contrast	rd-cluster shade	rd-sum entropy
	T1pc-maximum probability	Rsquare-dissimilarity	ak-cluster prominence	T1pc-sum average
	T1pc-average	Rsquare-IDMN	rk-sum entropy	T1pc-cluster shade
